# Single-Cell TCR and Transcriptome Analysis: An Indispensable Tool for Studying T-Cell Biology and Cancer Immunotherapy

**DOI:** 10.3389/fimmu.2021.689091

**Published:** 2021-06-07

**Authors:** Anna Pasetto, Yong-Chen Lu

**Affiliations:** ^1^ Department of Laboratory Medicine, Division of Clinical Microbiology, ANA FUTURA, Karolinska Institutet, Stockholm, Sweden; ^2^ Department of Pathology, University of Arkansas for Medical Sciences, Little Rock, AR, United States; ^3^ Winthrop P. Rockefeller Cancer Institute, University of Arkansas for Medical Sciences, Little Rock, AR, United States

**Keywords:** single cell, cancer immune, tumor microenviroment (TME), TCR - T cell receptor, immunotherapy

## Abstract

T cells have been known to be the driving force for immune response and cancer immunotherapy. Recent advances on single-cell sequencing techniques have empowered scientists to discover new biology at the single-cell level. Here, we review the single-cell techniques used for T-cell studies, including T-cell receptor (TCR) and transcriptome analysis. In addition, we summarize the approaches used for the identification of T-cell neoantigens, an important aspect for T-cell mediated cancer immunotherapy. More importantly, we discuss the applications of single-cell techniques for T-cell studies, including T-cell development and differentiation, as well as the role of T cells in autoimmunity, infectious disease and cancer immunotherapy. Taken together, this powerful tool not only can validate previous observation by conventional approaches, but also can pave the way for new discovery, such as previous unidentified T-cell subpopulations that potentially responsible for clinical outcomes in patients with autoimmunity or cancer.

## Introduction

### T-Cell Receptor

A T-cell receptor (TCR) is a heterodimer consisting of two chains, TCRα and TCRβ chains, that allow the recognition of peptides in the contest of major histocompatibility complex (MHC) molecules. Each of the two chains is made of a variable region and a constant region that are spliced together during the T cell development that happens in the thymus. In TCRβ chain, there are two constant region gene segments, Cβ1 and Cβ2, with some shared sequences. In TCRα chain, there is only one constant region gene segment, Cα. The variable region of the β chain consists of three gene segments called variable (V), diversity (D) and junctional (J), but the α chain only consists of the V and J segments. In human, 42 V segments, 2 D and 12 J are identified in β chain locus; and 43 V and 58 J for the α locus. Within each V segment, there are three hypervariable regions, or complementarity-determining regions (CDR1, CDR2 and CDR3). While CDR1 and CDR2 are encoded by the V segment, the CDR3 regions results from the juxtaposition of the V, (D) and J regions during somatic recombination. The joining of the V(D)J regions is imprecise, and nucleotides can be lost or added (e.g. the P and N nucleotides) during the process, resulting in a unique and unpredictable amino acid sequence for each CDR3 (1). It is clear that the structure of the TCR allows for great variability, which is further increased by the heterodimeric pairing of the α and β chains. It is estimated that the total number of possible combination could be greater than 10^18^ (2). The great variability of TCRs is essential to enable their unique ability to recognize antigenic targets, either pathogens or tumor cells. Lastly, the process of antigen recognition is also complicated. It relies on multiple interactions. The TCR needs to contact the MHC molecule on the cell surface, mostly by specific interactions with CDR1 and CDR2. The TCR also interacts with the peptide presented by the MHC molecule, mostly by specific interaction with the CDR3.

In the field of cancer immunotherapy, the identification not only of cancer antigens, but also of the antigen-specific TCRs, is a major research topic. Despite the evidence of tumor-specific T cells in cancer patients both among the tumor infiltrating lymphocytes (3–5) and in the peripheral blood (6–9), the presence of these cells is often not sufficient to induce cancer regressions even after checkpoint immunotherapy (10, 11). The reasons for these mixed clinical results are still not fully elucidated and cannot be addressed with a simple explanation. Nevertheless, it is commonly hypothesized that such antigen-specific T cells display an exhaustion phenotype that cannot easily be reverted (12), especially in the contest of an immunosuppressive tumor microenvironment (13). Adoptive cell therapy can potentially overcome these limitation by both increasing the number of cancer-specific T cells *ex vivo* before reinfusion and also by engineering these T cells with more powerful TCRs (14). The genetic transfer of TCRs requires the identification and isolation of powerful and specific TCRs. As described earlier, TCRs are heterodimers and only the match between the correct TCRα and β chain would enable a specific antigen recognition. TCR pairing is therefore one of the major challenges in the process of TCR identification.

Several approaches have been proposed to overcome the challenge of TCR pairing. Once a population of reactive T cells is identified, next-generation sequencing of bulk TCR clonotypes can provide a list of dominant TCRα and β clones that could be then paired accordingly to their frequency (15, 16). This approach gives the best results when the population of interest is fairly oligoclonal (most dominant TCRβ clonotype ≥ ~20%), but it is possible that the most dominant clonotypes need to be paired with each other using a matrix before the correct match is found. Another method that has been utilized to match TCRα and TCRβ chains from a bulk T-cell population is the Pairseq from Adaptive Biotechnologies (17). This approach is also based on next generation sequencing of both TCRα and TCRβ chains from a T cell subset, but the pairing of the chains is assigned with a statistical algorithm. The last approach is TCR sequencing at the single-cell level. This represents the best approach because it allows to quickly identify the correct TCRα and β pairs from each single cell present in a T-cell population of interest. Several different technical approaches have been utilized for single-cell TCR sequencing. In the following sessions, we will describe these robust and successful methods.

## Single-Cell TCR and Transcriptome Sequencing

### Step One: The Isolation of Single T Cells

**Figure 1:**
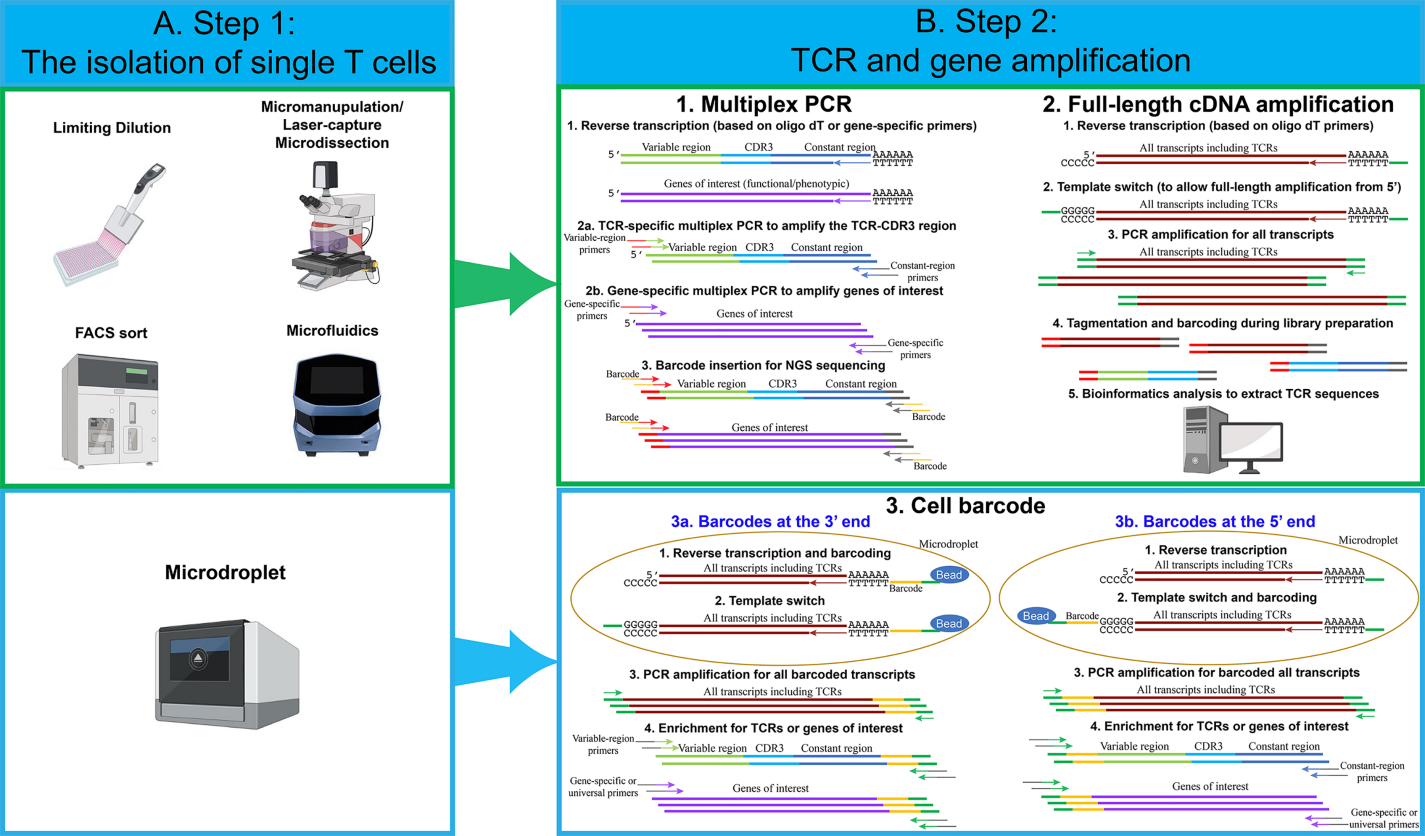
An overview of single-cell isolation techniques, followed by TCR and gene amplification strategies. **(A)** Several techniques have been used to isolate single cells. The most frequently used techniques are FACS sort and microdroplet techniques. For limiting dilution, micromanipulation, FACS sort and microfluidics techniques, multiplex PCR and full-length cDNA amplification approach can be used to perform single-cell TCR and transcriptome sequencing. For the microdroplet technique, cell barcode approach is used to perform single-cell TCR and transcriptome sequencing. **(B)** For the multiplex PCR approach, individual single cells are lysed in individual PCR tubes or wells. Reverse transcription is performed using oligo dT or gene-specific primers. Two PCR reactions are performed in individual wells using TCR or gene-specific primers. Notably, for each PCR reaction, approximately 70 variable-region forward primers are required to amplify the majority of TCRs. Two constant-region reverse primers are required, including one primer for constant-region Cα and one primer for constant-region Cβ1 and Cβ2. Lastly, barcodes for individual wells are added by an additional PCR reaction. Single-cell PCR products from individual wells are pooled and sequenced. For the full-length cDNA amplification approach, individual single cells are lysed in individual PCR tubes or wells, and reverse transcription is performed using oligo dT. All transcripts, including TCRs and genes of interests, are amplified by PCR reactions. Full-length cDNA products are cut into small fragments by tagmentation. Barcodes for individual wells are added by an additional PCR reaction. Single-cell PCR products from individual wells are pooled and sequenced. Bioinformatic analysis is used to extract TCR sequences and calculate the expression levels for genes of interests. For the cell barcode approach, single cells are lysed in individual microdroplets, and the cell barcodes for individual single cells are added either at the 3’ or at the 5’ end of the transcripts. For 3’ barcoding, barcodes are added at the reverse transcription step. After reverse transcription and template switch, all single-cell transcripts are pooled and amplified. Similar to the multiplex PCR approach, a pool of about 70 variable-region forward primers are required to amplify TCRs and genes of interest. Lastly, PCR products are sequenced and analyzed. For 5’ barcoding, barcodes are added at the template switch step. After barcoding, all of the single-cell transcripts are pooled and amplified. Unlike 3’ barcoding, only two constant-region reverse primers are required for each PCR reaction. Lastly, PCR products are sequenced and analyzed. Notably, for both 5’ and 3’ barcoding, tagmentation and PCR amplification by universal primers can be utilized, in order to analyze all transcripts and obtain whole-transcriptome data.

The first step in each single-cell sequencing technology is the isolation of single cells (**Figure 1A**). The conventional technique developed to isolate single T cells to obtain clonal T cell lines is called limiting dilution. This approach is relatively simple. However, due to the statistical distribution of cells per well, it is not very efficient. Typically, only one third of the wells contain a single cell when starting with a concentration of 0.5 cells per aliquot (18). Micromanipulation is another technique developed mainly to isolate embryos or stem cells, but it could be applied to T cells, particularly since the potential of generating human induced pluripotent stem cells to differentiate into anti-tumor T cells has been explore (19). A microscope-guided capillary pipette is used to pick single cells from a suspension culture (20). Laser-capture microdissection is similarly used to isolate individual cells or cell compartment from solid-tissue samples, such as biopsies, paraffin-embedded or cryo-fixed tissues (21–23). The main limitation with these approaches is that they are low-throughput and time-consuming.

To overcome such limitation, several approaches have been developed. One approach that has been commonly used is fluorescence-activated cell sorting (FACS), where the T cells are isolated based on the staining of pMHC multimers (8) or surface markers, such as PD-1 (15) or CD137 (16). This methodology allows to choose a specific population of interest but has the requirement of a high number of cells as starting materials. Microfluidic isolation of cells has the advantage of low sample consumption. When performed in closed systems, it also reduces the risk of contamination (24). The commercial platform Fluidigm C1 is an example of automated system for single cell capture coupled with cell-lysis, RNA extraction and cDNA synthesis. A more recent commercial system, the Chromium Controller from 10X Genomics, has recently gained popularity. The system is based on microdroplets, where cells are captured in aqueous droplets dispersed in oil phase. This system enables the isolation of tens of thousands of single cells simultaneously with high throughput and high capture efficiency (25). Notably, in the majority of experiments, no special modifications are needed for isolating single cells from T cells, compared to other cell types. However, because of the relatively smaller size of T cells, the microfluidics technique needs to be adjusted accordingly. For example, T cells can only be captured by the smallest, 5-10 µm integrated fluidic circuits (IFCs) using a Fluidigm C1 system.

### Step Two: TCR and Gene Amplification

The next step after single T-cell capture involves in the reverse transcription and amplification of TCR and/or genes of interest. In the following section, we describe the most common strategies for single-cell analysis (**Figure 1B**).

#### Multiplex PCR

The very first methodology developed to sequence TCRα and TCRβ chains was based on multiplex PCR followed by Sanger sequencing of the different amplicons (26). Although useful for the isolation of specific TCR clones, this methodology did not have the adequate throughput capacity to give an estimation of the TCR diversity in an T cell population. Only after the technical break-thought of multiple parallel sequencing (also called “next-generation sequencing” or NGS), it became possible to obtain a comprehensive knowledge of the TCR arrangement including V–J segments and the complete CDR3 sequence. A simple but effective approach to amplify the TCRs consists in a multiplex PCR where a pool of forward primers complementary to the different V segments of the TCRs and either a pool of reverse primers complementary to the different J segments or two reverse primers complementary to the C regions. The J segment primers are mainly utilized when TCR sequences are amplified from genomic DNA due to the intronic sequences. It’s possible to amplify TCR sequences from cDNA using the same pool of primers (27). However, only cDNA, but not genomic DNA, can be amplified using reverse primers complementary to the constant regions (28). Subsequently, additional genes associated with specific T cell functions (*e.g.* cytokines) can be also amplified in the same reaction. The introduction of short nucleotides, or barcodes, during the PCR reaction, makes it possible to pool the different amplicons and perform high-throughput sequencing by NGS (29). More recently, the technological developments have resulted in mainly two methods commonly used to perform the amplification of the single cell transcriptome that can be divided into full-length cDNA amplification and cell barcode approach.

#### Full-Length cDNA Amplification

This approach generates a sequencing library separately for each single-cell transcriptome. While it is more expensive than targeting specific genes, it has the benefit of broader data collection (*e.g.* on isoforms, etc.). The full-length approach has also been used to identify TCR sequences for several applications, including TCR repertoire analysis and pairing of TCRα and β chains. This approach is often used when the single cells are captured in individual wells, for example, after FACS sort or when captured by a microfluidic device. After cell lysis, the mRNA molecules are reverse transcribed using oligo dT primers at the 3´end. A universal sequence is added at the 5´end by a template-switch strategy. The template switch strategy is usually employed when there is a variation about the exact sequence of a gene, such as TCR variable region, or when we intend to amplify all transcripts. The strategy employed is based on the particular behavior of the reverse transcriptase that adds a stretch of non- template dCTPs at the 3′ end of the cDNA. This stretch of dCTPs can bind to a specifically designed oligo that contains a complementary stretch of poly-G followed by a universal sequence (30).

Once the universal sequence is introduced at the 5´end of each transcript, the full-length transcript (from the 5’ to the 3’ end) can be amplified. The amplification step is followed by a “tagmentation” step, usually using a transposase that can insert Tag sequences that are then used to insert barcodes. The libraries prepared with this method are not enriched for the TCR sequences. Therefore, to extract each TCR sequence, it is necessary to use a bioinformatic tool. For TCRs, the traditional reference-based assembly, where the sequences obtained are compared to a reference genome, is combined with *de novo* assembly for the CDR3 region that has to be reconstructed based only from the actual sequences. Several tools have been developed to perform this type of analysis. An example is TraCeR, a computational method that allows to reconstruct the variable sequence of TCRα and TCRβ chains through use of a “combinatorial recombinome” library of all possible TCR sequences, this method was initially used in combination with the FluidigmC1 System (31, 32). Another computational method is “single-cell TCRseq” (33) that employs several consecutive steps to first identify and count RNA reads mapping to specific TCR V and C regions, then perform multiple alignments to create consensus V and C gene sequences. Finally, gaps in the sequence are filled similarly to *de novo* transcript assembly. A similar multistep approach is also used by TRAPeS (TCR Reconstruction Algorithm for Paired-End Single-cell) (34). In this software, the V and J segments are first identified for each chain. Subsequently, a set of putative CDR3 reads are identified as potential match to the one from the previously identified V and J segments. Lastly, an algorithm is used to reconstruct the CDR3 region from the putative CDR3 reads. We also utilized a similar approach to assemble TCR sequences (35). The TCR sequence reads were first aligned to V segments, and then TCR reads with identical CDR3 region sequences were merged to assemble the full-length TCR sequences. Lastly, VDJ Puzzle is a useful tool that allows to reconstruct the TCR sequence from single cell transcriptome data (36). This method was first described to link the TCR sequence from antigen-specific cells based on their gene expression profile.

#### Cell Barcode

The cell-barcode strategy adds cell barcodes, about 10-20 bp random nucleotide sequences, to individual single cells. This makes it possible to pool all transcripts coming from thousands of cells, increasing the throughput and decreasing the cost dramatically (17, 37). This approach is usually employed after each cell has been captured into a microdroplet and lysed. In addition to cell barcodes, molecular barcodes are often added at the same time. Molecular barcodes, also known as unique molecular identifiers (UMIs), are about 10 bp random nucleotide sequences, which allow us to identify each individual molecules/transcripts. The advantage of UMI technique is that the UMI counting will not be altered even after imbalanced PCR amplification.

For the cell barcodes at the 3’ end, all mRNA transcripts present in the cells are reverse transcribed using oligo dT primers containing both the cell barcodes and molecular barcodes. Next, the template switch strategy is used to add a universal sequence at the 5’ end. This enables the PCR amplification of all transcripts. Lastly, a set of variable-region forward primers and gene-specific forward primers are utilized to enrich TCRs and other genes of interest. An additional PCR reaction is required to add necessary DNA sequences for next-generation sequencing.

For the cell barcodes at the 5’ end, all mRNA transcripts are reverse transcribed using oligo dT primers, but the cell barcodes and molecular barcodes are added at the 5’ end during the template switch step. Next, all transcripts are pooled and amplified by PCR. TCR and other genes of interest can be amplified by constant-region reverse primers and gene-specific reverse primers. Lastly, an additional PCR reaction is used to add necessary DNA sequences for next-generation sequencing. Notably, the whole-transcriptome analysis can be achieved by both 5’ and 3’ end barcoding. After the PCR amplification and an additional tagmentation step for all transcripts, transcripts at the 5’ end or 3’ end can be processed and sequenced. Because cell barcodes and molecular barcodes are required to be retained in the entire process, only the gene sequences near the 5’ or 3’ end, approximately 200-500 bp, can be sequenced. As the results, the information of full-length transcripts, including isoforms, is lost using this strategy.

The strengths and weaknesses of different strategies are summarized in **Tables 1** and **2**. In recent years, the single-cell field has been in favor of the microdroplets with cell barcode approach, because a higher number of cells can be obtained, compared to other approaches. Although the microdroplet approach is less sensitive to detect low abundant genes, this concern is outweighed by the high cell numbers and robust bioinformatic tools. In addition, microdroplets with barcodes at 5’ end can use a minimum number of primers for TCR amplification, compared to barcodes at 3’ end. As a result, 5’ barcoding is more suitable for T-cell studies that require TCR sequence information, such as clonality analysis.

**Table 1:** Comparison between multiplex PCR approach and full-length cDNA amplification approach.

Multiplex PCR	Full-length cDNA amplification
More sensitive for individual genes or TCRs	Less sensitive
Lower cost	Higher cost for deeper sequencing
A set of ~70 primers for TCR variable region is required*	Only a set of universal primers is required*
Impossible to obtain whole-transcriptome data	Available whole-transcriptome data
Impossible to obtain full-length TCR sequences	Available full-length TCR sequences

*Another set of primers is required for nested PCR amplification.

**Table 2:** Comparison between cell barcodes at the 5’ end and 3’ end.

Barcodes at the 3’ end	Barcodes at the 5’ end
More efficient to add barcodes	Less efficient to add barcodes
A set of ~70 primers for TCR variable region is required*	Only 2 primers for TCR constant region are required*
Suitable for all types of cells	Only suitable for TCR and BCR studies
More kits and applications available due to popularity	Less kits and applications available

*Another set of primers is required for nested PCR amplification.

## Spatial Transcriptomics

**Figure 2:**
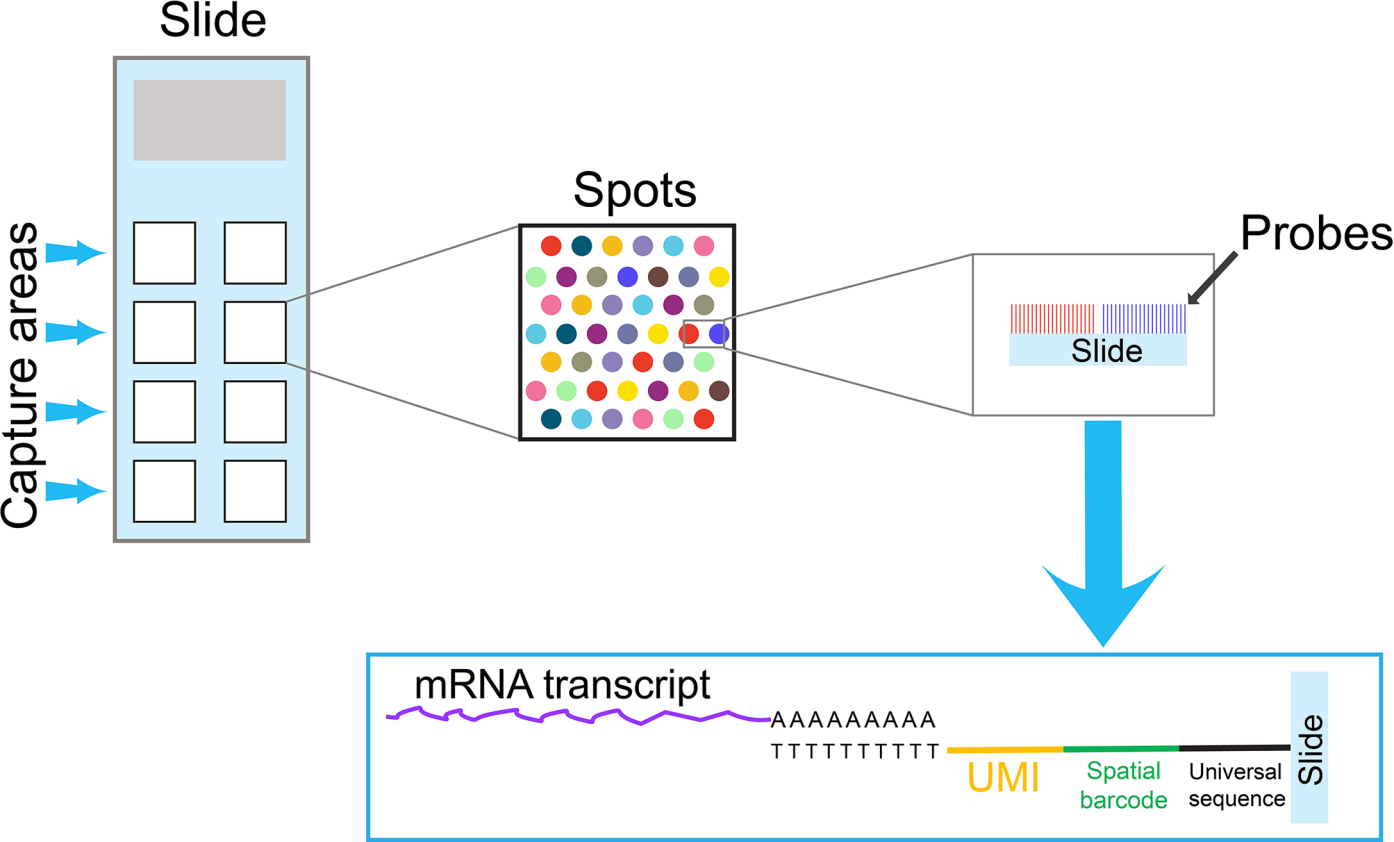
An overview of a spatial transcriptomics technique. A specialized slide contains several capture areas. Each capture area contains thousands of spots, and each spot is coated by oligo dT-based probes. To obtain spatial information, mRNA transcripts from a tissue section are captured by these oligo dT-based probes on spots. Because probes on each spot contain a unique spatial barcode and UMIs, the spatial information can be preserved in the subsequent PCR reactions. Similar to the single-cell cell barcode approach with 3’ barcoding, transcripts containing spatial barcodes at the 3’ end are amplified by PCR reactions and sequenced. The data obtained from spatial transcriptomics can combine with single-cell transcriptome data to obtain comprehensive information for cell-cell interactions in the tissue microenvironment.

Single-cell samples are often prepared by enzymatic or mechanical dissociation. As a result, spatial information is lost during the sample preparation. However,the interactions between T cells and the adjacent cells in the tumor microenvironment may influence the transcriptome of individual T cells. Stahl PL et al. have developed a new technique to provide two-dimensional, spatial information, which can complement single-cell transcriptome data analysis (38). In this technique, mRNA transcripts from a tissue section are captured on an array by oligo dT-based probes, which contain spatial barcodes and UMIs (**Figure 2**). Similar to the single-cell transcriptome analysis with barcodes at 3’ end, transcripts containing barcodes at 3’ are amplified and sequenced. This technique has improved significantly in recent years, and it can now reach near the single-cell resolution, at approximately 1-10 cell resolution per spot, depending on the tissue type. In addition, spatial information can combine with traditional immunofluorescence staining to detect both mRNA and protein expression at the same time.

Since the initial publication, scientists have used this spatial transcriptomics technique on a variety of tissue specimens. We have identified two publications related to T-cell studies. Thrane K et al. utilized this technique to study melanoma lymph node biopsies, and they were able to visualize the transcriptional landscape within the tissue (39). The lymphoid area in close proximity to the tumor region showed a specific expression pattern, which might reflect the unique feature in tumor microenvironment. Notably, an IFN-γ gene signature, likely from activated T cells, was identified within the transition area between melanoma and lymphoid areas. In another study, Ji AL et al. combined the techniques of spatial transcriptomics, single-cell transcriptome and multiplexed ion beam imaging to study the architecture of cutaneous squamous cell carcinoma (40). In addition to a tumor-specific keratinocyte population that they identified, they also observed regulatory T cells co-localized with CD8^+^ T cells in the compartmentalized tumor stroma. Taken together, spatial transcriptomics may have significant potential in the future study.

## T-Cell Neoantigen Identification

Cancer is caused by a series of genetic alterations that occur in normal cells and are responsible for their transformation in malignant cells. These alterations confer an advantage to the affected cells, such as increased proliferation and inhibition of apoptosis. However, these can also result in the production of mutant proteins that are immunogenic and can be targeted by the immune-system. When such mutated proteins become targets for the immune-system, they can be called neoantigens. Because neoantigens are not expressed by normal cells, they represent attractive targets for cancer therapy. In the vast majority of cases the identified neoantigens arise by single amino-acid substitutions. The mutated peptides can be processed and presented by MHC molecules, and then the peptide/MHC complexes can be recognized by T cells. The presence of neoantigen-reactive T cells have been identified across different cancer histology, like lung cancer (41, 42) bladder cancer (43), head and neck cancer (44, 45), ovarian cancer (46–49) pancreatic cancer (50, 51) and gastrointestinal epithelial malignancies (35, 52–54). Interestingly, the T cells identified in these studies recognized unique somatic mutations, with few exceptions where the T cells recognized hot spot mutations on oncogenes, like KRAS (55, 56) and p53 (9, 47, 48, 54). Additional studies are needed in order to evaluate systematically the immune-response against hot spot mutations in these highly valuable targets.

Single cell sequencing is a powerful tool for T cell biology discovery and can be employed to dissect specific functional and phenotypical signatures. T cells have the ability to recognize specific antigens in the contest of MHC molecules, and this ability can be harnessed to develop anticancer therapies, therapies against autoimmune diseases and antiviral therapies. The “holy grail” of T cell immunology would be to predict the antigen-specificity of a T cell simply by studying its TCR sequence and structure. Although this antigen-prediction is not available yet, several technologies have been developed to identify an epitope recognized by a given T cell and rapidly isolate its TCR. In the following sections we will describe some of the most successful approaches used to identify T cell antigens and their specific TCRs.

### pMHC Multimers

One of the most common approaches used for this purpose is based on the capacity of T cells to bind pMHC multimers. If the multimers are labelled with fluorescent probes, the T cells can be identified and isolated by flow cytometry (57). This strategy can only be applied when the target epitope is known and also suitable to be presented on pMHC multimers. Typically class I epitopes give more specific binding than class II. Despite these limitations this approach has been effective to discover important cancer antigens that could be used for immunotherapy (58, 59). A more recent version of this strategy employs pMHC multimers labelled with DNA barcodes [TetTCR-Seq (60)] which has the advantage of high-throughput and the possibility to integrate single-cell transcriptomics, T cell phenotype and TCR sequence isolation. To address specific binding and recognition of class II restricted epitope, Graham DB et al. developed a high-throughput approach for screening of DNA-encoded pMHC class II libraries to provide functional recognition by TCRs identified from single cell sequencing (61). Additionally, DNA-barcodes were linked to magnetic nanoparticles, as described by Peng et al. (62), to identify CD8^+^ neoantigen-specific T cells from tumor and blood samples of melanoma patients. This last study highlights how both the antigen-binding specificity and the sensitivity in detecting rare T-cell populations is important to identify reactive T cells from clinical samples.

### Screening of Antigenic Libraries

In the previous section, we described examples of technologies that enabled to isolate specific TCRs for known antigens. This type of approach is very useful when the specific antigen and its MHC-binding epitope is known, for example when targeting viral antigens or shared cancer antigens (both mutated and normal proteins). A different situation is represented by T cells and TCRs that have been isolated based on some particular characteristic (*e.g.* the expression of a specific marker or their high frequency in a particular T cell subset), but their specificity is unknown. There are several strategies that can be used to identify the cognate peptide for orphan TCRs (63). Most approaches are based on empirical testing where the T cell activation status is evaluated after co-culture with the candidate antigens (15, 16, 64). An interesting variation of these approaches consists in the screening of pMHC libraries, where the TCRs are isolated based on their affinity to the different pMHC, but without knowing the antigenic specificity (63).

## Single-Cell Studies on T-cell Biology and Cancer Immunotherapy

**Figure 3:**
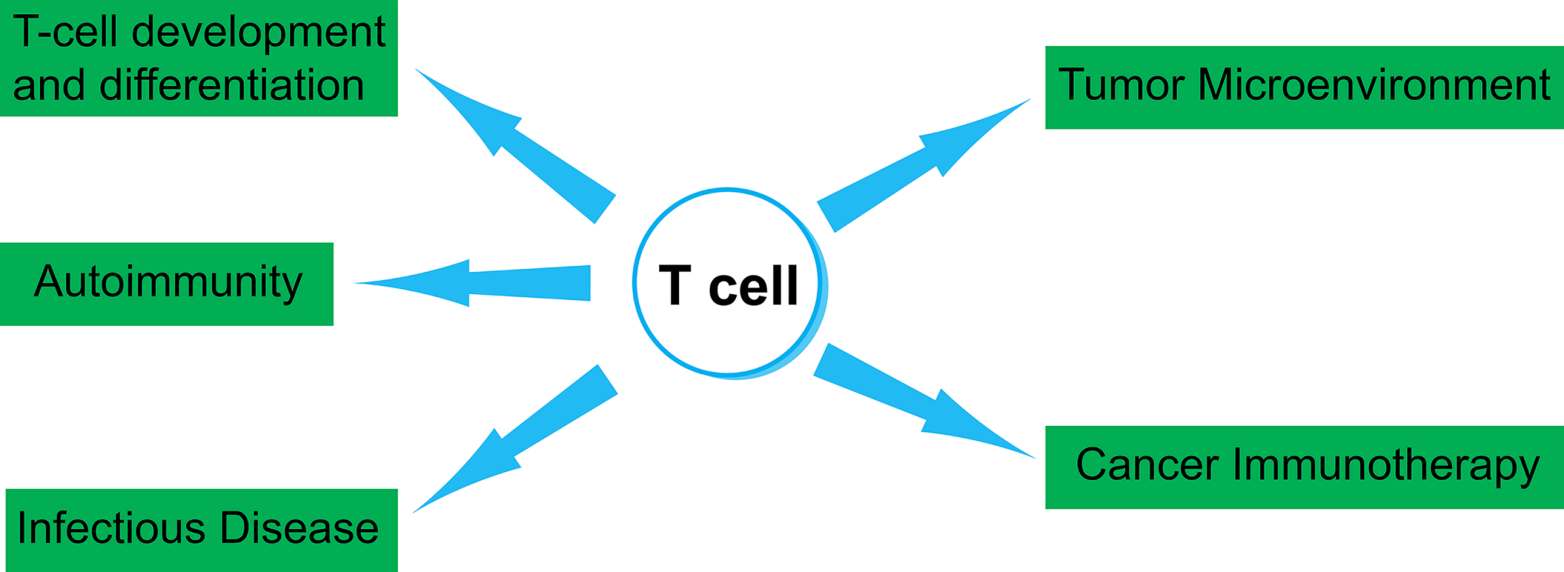
Several aspects of T-cell biology that can be studied by single-cell techniques. In this review article, we summarize studies that utilized single-cell TCR and transcriptome analysis. Those studies include fields in T-cell development and differentiation, autoimmunity, infectious disease, tumor microenvironment and cancer immunotherapy.

Single-cell transcriptome analysis has been used to study the biology of T cells in several areas, including T-cell development, differentiation, and responses during infection and autoimmunity. The role of T cells in tumor microenvironment and cancer immunotherapy is also a topic for intensive studies (**Figure 3**). Single-cell TCR analysis can also provide important information for these studies, such as TCR pairing and clonality. Furthermore, it has been demonstrated that combining TCR and gene expression information can provide deeper understanding for T cell-mediated immune responses (29). Because many high-quality manuscripts using single-cell techniques have been published in recent years, we would like to focus our discussion on some of outstanding publications utilizing single-cell transcriptome data alone or together with the TCR sequencing data. Notably, high-dimensional flow cytometry or mass cytometry (CyTOF) can investigate over a dozen of cell-surface markers at the single-cell level (65). For the scope of this article, we will not discuss findings generated by this technique.

## T-Cell Development and Differentiation

The thymus is the key organ for T cell development. Abnormalities of T-cell development, including positive and negative selections, can lead to autoimmune diseases (66, 67). Park JE et al. performed a comprehensive single-cell study on prenatal and postnatal thymus samples, including adult samples (68). Pseudo-time analysis showed that gene markers and trajectory for T cell development were consistent with previously knowledge in mice (69). However, the authors also identified a previous unknown subset, GNG4^+^ CD8αα^+^ T cells in the thymus. This subset of T cells could fully mature into a CD8A^high^/CD8B^low^ phenotype, but T cells from the mouse counterpart could become triple negative (CD8A^low^ CD8B^low^ CD4^low^) cells.

T cells can further differentiate in the peripheral tissue. Li N et al. utilized single-cell sequencing and other techniques to characterize CD4^+^ T cell compartment in the human fetal intestine (70). Additionally, through the single-cell trajectory analysis, the authors observed the generation of memory-like CD4^+^ T cells in the human fetal intestine. In another report, Galletti G et al. used single-cell analysis to study human CD8^+^ memory T cells from peripheral blood under physiological conditions, and identified two previously unrecognized subsets of stem-like CD8^+^ memory T cells (71). The PD-1^-^ TIGIT^-^ subset was committed to a functional lineage, whereas the PD-1^+^ TIGIT^+^ subset was committed to a dysfunctional, exhausted-like lineage. Lastly, using the transcriptome and TCR sequencing analysis, Patil V et al. identified the CD4^+^ cytotoxic T cell population within the T_EMRA_ (effector memory T cells expressing CD45RA) subset (72). In addition, they could identify four distinct subsets within the CD4^+^ cytotoxic T cell population, based on single-cell transcriptome analysis. These studies provide insights on the potentially durable immunity generated by T cells.

### T-Cell Biology in Autoimmunity

T cells play an important role in autoimmunity. Corridoni D et al. utilized single-cell transcriptome analysis to study colonic CD8^+^ T cells in health and ulcerative colitis, an inflammatory bowel disease (73). They found that IL-26 was expressed in terminally differentiated, dysfunctional CD8^+^ T cells from ulcerative colitis. Human IL-26 could attenuate immune responses in a mouse model of acute colitis. Next, Strobl J et al. used single-cell technique to study tissue-resident memory T cells in skin, and they identified RUNX3 and LGALS3 as new markers for this type of T cells (74). They also identified a large number of host-derived tissue-resident memory T cells in skin lesions from patients developing graft-versus-host disease, suggesting the potential contribution of these cells to this disease. Lastly, Seumois G et al. studied the roles of CD4^+^ T helper cells and regulatory T cells in patients with asthma, and they identified CD4^+^ T cell subsets that might contribute to the pathogenesis of allergy and asthma (75).

### The Role of T Cells in Infectious Diseases

Single-cell analysis has become a powerful tool to analyze T-cell responses during the infection. For example, Kazer SW et al. studied peripheral blood mononuclear cells from four individuals with acute HIV infection, and they discovered gene response modules that were different between cell subsets and were changed during the course of the infection (76). More importantly, during COVID-19 pandemic, several studies utilized single-cell analysis to study T cells from COVID-19 patients (77–82). In one of the studies, abundant exhausted T cells with skewed TCR repertoire were found in the immune landscape of severe COVID-19 patients (79). In another study, single-cell sequencing was performed on immune cells isolated from cerebrospinal fluid (CSF) in COVID-19 patients with neurological sequelae (82). Those CSF T cells showed a reduced interferon response compared to viral encephalitis.

### T-Cell Biology in Tumor Microenvironment

Single-cell technique has been used insensitively to study T-cell biology in tumor microenvironment. T-cell transcriptome profiles in the majority of cancer types have been published (32, 83–91). Li H et al. studied intra-tumoral T cells isolated from 25 melanoma patients (92). They discovered a significant portion of the CD8^+^ T cells were in a gradient of “transitional” states, between a healthy/cytotoxic T-cell state and a dysfunctional T-cell state. In addition, T cells in the dysfunctional state still had proliferative capacity and formed large T-cell clones. In another study, Ghorani E et al. utilized single-cell analysis and high-dimensional flow cytometry analysis to analyze non-small cell lung cancer specimens (93). They found the correlation between T-cell differentiation status and tumor mutational burden. The authors proposed that the characterization of intratumoral T cells might help to predict the outcome of immunotherapy. Lastly, Oh DY et al. studied T cells isolated from bladder cancer and identified several subsets of CD4^+^ T cells containing gene signatures for cytotoxic T cells.

### T Cells and Cancer Immunotherapy

Investigators have utilized single-cell techniques to study intratumoral T cells prior and after checkpoint immunotherapy for melanoma (94, 95). One of the important findings was the identification of a CD8^+^ T cell subset that expressed TCF7, a key transcription factor for “memory-like”, proliferation-competent, exhausted T cells (96–98). In addition, the presence of TCF7^+^CD8^+^ T cells could predict clinical response to checkpoint immunotherapy. Next, Luoma AM et al. utilized single-cell analysis to study T cell populations in colitis, a common and severe side effect of checkpoint immunotherapy (99). They observed a substantial fraction of colitis-associated CD8^+^ T cells that were likely originated from tissue-resident populations, identified by single-cell TCR clonality analysis. Similarly, studies were carried out to perform single-cell TCR/transcriptome analysis on peripheral blood T cells after checkpoint immunotherapy (91, 100). This approach was able to identify genes associated with clinical responses as a result (100).

Chimeric antigen receptor (CAR) T cell therapy has shown dramatical clinical responses against B-cell malignancies. The majority of the CAR designs utilized two different co-stimulatory domains derived from CD28 and 4-1BB molecules. Boroughs AC et al. attempted to use single-cell transcriptome analysis to identify gene signatures associated with different CAR designs (101). The authors identify a transcriptional signature shared between CAR designs, as well as a unique, distinct signature associated with 4-1BB co-stimulatory domain, compared to CD28 co-stimulatory domain. In another study, Sheih A et al. took advantage of highly-diverse, endogenous TCR sequences and utilized these sequences as natural barcodes (102). They were able to track CAR T cells after therapy and perform single-cell analysis by following these barcodes. Taken together, the results obtained by single-cell analysis provides more insights on how to improve the cell products for CAR T-cell therapy.

## Caveats on Experimental Design and Data Interpretation

Single-cell TCR and transcriptome analysis is a very powerful tool, but it can be very costly as well. We hope those outstanding publications described above can help readers to design single-cell experiments and acquire data that cannot be obtained by other approaches. One of the common errors is to utilized a single-cell sequencing approach even when the proposed research goals can be simply accomplished by “bulk” RNA-seq analysis, which not only costs less, but also can acquire higher quality of data, especially for low abundance transcripts.

Although the data quality of single-cell transcriptome has improved significantly in recent years, the single-cell data still suffer from the sensitivity issue for low abundance transcripts, also known as technical dropouts. Several computational algorithms have been developed to specifically address this issue for single-cell transcriptome analysis (103–105). However, the performance of these algorithms is still far from perfect, and the results may differ between algorithms (106). Therefore, researchers are still needed to beware of potential artifact and bias involved in the data analysis and interpretation. We still highly recommend researchers to validate the observations by another independent approach, such as flow cytometry or targeted sequencing.

Another important caveat is that the observations tend to be simplified, leading to binary thinking. The commonly used clustering technique in single cells analysis is based on the assumption that cells are defined into discrete populations, which might not reflect the true biology. Van der Leun et al. have proposed that T cells in the tumor microenvironment are in a gradient of cell states rather than discrete populations (107). Therefore, we should be cautious about data interpretation using the clustering technique.

## Future Perspective: Highly Personalized, T Cell-Based Cancer Immunotherapy

Studies utilizing adoptive cell transfer of tumor-infiltrating lymphocytes (TIL) have shown that this approach can result in durable and complete regressions of advanced cancer diseases, in particular metastatic melanoma. Very frequently, reactivities against neoantigens were present among the infused TIL (108–110). Despite the evidence of clinical responses, the adoptive transfer of neoantigen-reactive TIL has several limitations. The transferred cells are highly differentiated and can have a limited proliferative ability, leading to lack of persistence *in vivo* after adoptive cell transfer (111, 112). Additionally, because it is impossible to control the skewing of the T cell repertoire during expansion, the neoantigen-specific TIL could lead to low abundance in the infusion product. For the same reason, it is also very difficult to control the number and the quality of the neoantigen that are targeted. To overcome some of these limitations, the genetic transfer of neoantigen-specific TCRs has been proposed (113–115). With this approach, it will be possible to introduce highly specific TCRs into less differentiated cells, and to combine TCRs with several specificities, affinities and HLA restrictions in one infusion product, potentially increasing the possibility of clinical response (14). This approach has nevertheless its own challenges, which are mainly related to finding a reliable source of neoantigen-reactive T cells from where to isolate the TCRs, as well as rapidly and efficiently transferring the TCRs to new recipient cells for treatment.

In targeting unique somatic mutations by adoptive T-cell therapy, it is equally important to consider other aspects that may reduce the efficacy of the therapy. Tumor heterogeneity is a major obstacle not only because the targeted neoantigen may not be expressed on every cell, but also because the MHC elements may not be expressed uniformly or even lost (116, 117). Another factor to consider is that the T cell functionality may not be always optimal even in the presence of the neoantigen-specific TCR. Several reports have highlighted the dysfunctionality of exhausted T cells in cancer patients (118, 119). Therefore, a desirable therapeutic approach would target several neoantigens, possibly restricted to different HLA elements and would be carried out by the most effective T cells. Different strategies have been proposed to overcome some of the most important issues, such as the selection of T cell subsets with a stem-like phenotype to improve persistence and antitumor activity (120) or the genetic modification of T cells to secrete IL-12 in order to promote HLA expression and cross-presentation by surrounding cells in the tumor microenvironment (121).

In summary, the single-cell TCR and transcriptome analysis has enabled T-cell biologists to ask critical questions and obtain interesting findings. This newly available research tool may help us to improve the current immunotherapy and develop new treatments for cancer and other diseases. We look forward to more exciting discoveries in the coming years.

## Author Contributions

AP and YL contribute equally in writing and discussion. All authors contributed to the article and approved the submitted version.

## Funding

AP was supported by the Sjöberg Foundation, Region Stockholm Centrum För Innovativ Medicin (CIMED), Svenska Läkaresällskapet, Cancerfonden, Ruth och Richard Julins Stiftelse. YL was supported by funding from the Winthrop P. Rockefeller Cancer Institute.

## Conflict of Interest

The authors declare that the research was conducted in the absence of any commercial or financial relationships that could be construed as a potential conflict of interest.
